# Phase-based design of CO_2_ capture, transport, and storage infrastructure via *SimCCS*^*3.0*^

**DOI:** 10.1038/s41598-023-33512-5

**Published:** 2023-04-21

**Authors:** Zhiwei Ma, Bailian Chen, Rajesh J. Pawar

**Affiliations:** Earth and Environmental Sciences Division, Los Alamos National Laboratory, Los Alamos, New Mexico USA

**Keywords:** Energy storage, Carbon capture and storage

## Abstract

The design of optimal infrastructure is essential for the deployment of commercial and large-scale carbon capture and storage (CCS) technology. During the design process, it is important to consider CO_2_ capture and storage locations and CO_2_ transportation pipelines to minimize the total project cost. *SimCCS*, first introduced in 2009, is an integrated open-source tool to optimize CCS infrastructure. The core CCS infrastructure design problem in *SimCCS* is structured as a mixed-integer linear programming problem by selecting the optimal pipeline routes, searching CO_2_ source capture and storage locations, and determining the corresponding CO_2_ amounts to meet desired capture targets. Multiple important and practical features have been developed to the latest version of *SimCCS*, *SimCCS*^*3.0*^. One of these features is phase-based modeling which enables users to dynamically design the CCS infrastructure. We demonstrate the phased-based modeling capability using two CCS infrastructure optimization case studies. The results from these case studies reveal that the phase-based modeling capability in *SimCCS* is particularly useful to optimize the dynamic deployment of CCS projects.

## Introduction

Carbon capture and storage (CCS) refer to the capturing and then storing CO_2_ into subsurface formations, such as saline aquifers, depleted oil/gas reservoirs, and coal seams^1^. CCS is an important technology to reduce CO_2_ emissions from various sources to the atmosphere^2^ and it is essential for clean energy transition to alleviate climate change by limiting global warming to 2 °C. In 2018, the U.S. Congress enacted the Bipartisan Budget Act of 2018^3^, in which the US Internal Revenue Code Section  45Q was reformed^4^. Section 45Q indicates that operators can claim tax credits if storing CO_2_ in saline and other forms of geologic storage and using CO_2_ for enhanced oil recovery. The 45Q tax credit can only be claimed for 12 years once a facility is placed in service^4^. However, commercial-scale CCS projects are expected to potentially last longer than 12 years, for example, a CarbonSAFE project in US will last for 30 years. To consider this time-varying 45Q credits in CCS modeling, it is also necessary to develop phase-based CCS design tool to consider the 45Q tax credits.

Additionally, a certain CCS project often evolves multiple CO_2_ point sources from various types of facilities, such as power plants, natural gas processing plants, hydrogen plants, and ethanol plants. In practice, not all the capturing facilities start operating at the same time and lasting for the entire project life. For example, some new facilities may become available after certain years due to delay in construction or deployment and some coal power plants may gradually be shut down and phased out. Specifically, as indicate by Climate Analytics, the global coal-fired power generation should be reduced and phased out before 2040^5^. This information can be obtained or anticipated before designing CCS project deployment. Including this prior information into CCS infrastructure design process would improve the effectiveness and feasibility of CCS design. Therefore, a new capability to model various scenarios of source status for CCS operations is required, which is another motivation of this work.

The successful deployment of any CCS project requires the proper design of CCS infrastructure related to CO_2_ point sources, storage sites, and pipeline networks^6^. *SimCCS*, an open-source CCS design tool developed by Los Alamos National Laboratory, is capable of performing CCS infrastructure designs to meet certain decarbonization targets^6–9^. *SimCCS* is one of the most used tools for CCS design optimization. Since first released in 2009, *SimCCS* has been applied to different types of CCS infrastructure development problems^10,11^. *SimCCS* is written in Java language and is an open-source CCS design tool and it is available to download, test, and further develop via GitHub. Although its feasibility and effectiveness of the recently released version of this tool^6^, have been demonstrated through various studies, further developments have to be made to solve more complex and challenging CCS deployment problems including phased-based modeling of CCS project design.

Here, we have developed and added a new capability in *SimCCS* tool to dynamically model CCS operation tool. The dynamic modeling capability entails the partition of the predefined project life into multiple time intervals and then performing of CCS infrastructure design by aggregating time-dependent quantities of interest together during optimization. This dynamic modeling module enables users to divide a CCS project into multiple phases and to define time-varying CO_2_ capturing targets according to their specific needs. Here, using the developed feature, we can simulate the evolution of CCS operations and deployments over time. Specifically, with the dynamic module in *SimCCS*, we can model two practical operational scenarios including the consideration of enhanced 45Q tax credits and dynamic evolution of CO_2_ point source open/closed status, which will be discussed in detail in this work.

A typical CCS infrastructure consists of CO_2_ capturing facilities or point sources, CO_2_ injection and storage facilities, and CO_2_ transportation pipeline networks that connect sources and sinks^12–15^. Before deploying any CCS project, it is essential to perform a systematical and proper CCS infrastructure design analysis, otherwise, many potential issues, e.g., higher costs and/or increased risks may be raised. The questions that need to be answered during the CCS infrastructure design include (1) how many tonnes of CO_2_ is captured from each CO_2_ point source, (2) where and how much the captured CO_2_ should be stored in geologic sinks, (3) where pipelines should be built to transport CO_2_ and what is the size and length of each pipeline segment, (4) how to determine optimum flow rates for CO_2_ transportation, etc. Because these questions are often inter-dependent, correlated and cannot be solved independently, it is necessary to integrate these CCS design questions into one optimization problem, in which each decision parameter can be determined by minimizing or maximizing a defined objective function with some constraints.

Optimization is a commonly used technique in energy resources productions^16–20^ and CO_2_ storage^21^. However, limited studies of the comprehensive optimization of CCS infrastructures related to the consideration of CO_2_ point sources, CO_2_ storage sinks, and the pipeline network that connects sources and sinks have been reported in the past. Examples of CCS infrastructure optimization can be found in several previous studies^13–15,22^. Although some previous studies have investigated the dynamic features for CCS infrastructure designs^7,13^, many of the existing studies did not consider the varying capture amounts from different sources and the dynamic evolution of statuses of open/closed for different sources and the enhancement of 45Q tax credits. In addition, the application of dynamic modeling in large-scale CCS project deployment design has rarely been explored in literature. Here, we fill this research gap by developing phased-based CCS infrastructure modeling.

**Figure 1:**
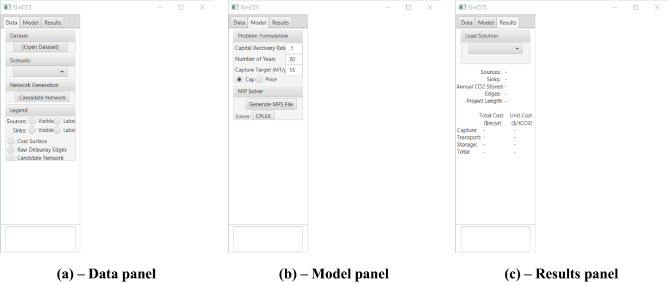
Different panels in *SimCCS*^*2.0*^.

As presented in Fig. 1. There are three panels available in *SimCCS*^*2.0*^, including (1) Data panel for importing and visualizing input data (sources, sinks, and candidate networks if generated) and for generating new candidate pipeline networks, (2) Model panel for selecting CSS project design scenario and for preparing the corresponding input files for optimization, and (3) Results panel for visualizing the optimized solutions in terms of pipeline construction and source/sink usages and providing quantitative analysis of CCS operations, such as unit cost for capturing, storing, etc. In *SimCCS*^*2.0*^, two CCS technology deployment analysis modules are available including the *Cap* module^8^ and the *Price* module^23^. The *Cap* module can be used to determine optimum CO_2_ infrastructure to meet a project CO_2_ capture target by minimizing the total project cost while the *Price* module can be used to determine whether the system-wide CO_2_ is emitted or is captured, transported, and stored according to a predefined price^6^.

The new version of *SimCCS* is *SimCCS*^*3.0*^^9^, which consists of four main components, i.e., *SimCCS*, NiCO_2_LE, SCO_2_T^24,25^, and CostMAP^26^. NiCO_2_LE provides input data related to CO_2_ point sources, e.g., source locations and corresponding capturing cost, SCO_2_T prepares input data related to the storage side including sink locations and injection costs, etc., CostMAP generates a realistic cost surface, which is then applied for the generation of candidate pipeline routes, and *SimCCS* is the core optimization engine to determine the optimum CCS infrastructures and deployments using the provided inputs from the previous three components. The CCS design optimization in *SimCCS*^*3.0*^ is represented as a mixed-integer linear programming (MIP) problem because of its advantages of flexibility, rigorousness, robustness, and extensive modeling capabilities for design optimization problems^27^.

The contribution of this study is summarized here. First, we have developed and implemented the dynamic modeling framework for CCS project design optimization and integrated the developed capabilities into the new version of open-source *SimCCS*^*3.0*^. Second, we investigate CCS infrastructure design optimization by including two practical operation and design considerations including potential enhancements to 45Q tax credits and dynamic evolution of CO_2_ point source status. Finally, we tested the developed methods via two case studies and the results demonstrate the feasibility, robustness, and effectiveness of our methods. The structure of this paper goes as follows: We will introduce the integration of dynamic modeling into *SimCCS*^*3.0*^ in Sect.  "Phase-based modeling". Next, we will test developed functionalities on two hypothetical case studies including a case study focused on Wyoming and a case study focused on the intermountain west (I-WEST) region of the US in Sect.  "Case studies". Finally, we will summarize the major findings of this work in Sect.  "Conclusion".

## Phase-based modeling

### Basic model formulations

The basic formulations presented here are adapted from several previous *SimCCS* studies^6,9,28^. For more detailed explanations of these formulations, those publications can be referred to. In this paper, we will briefly introduce the formulations and equations. The CCS infrastructure design problem can be formulated as a MIP problem, which involves an objective function, decision variables, and constraints. To implement phase-based CCS design, it is required to partition the entire project time horizon (${L}_{project}$) into multiple time intervals, and each interval can be represented as $t$ with the length of ${L}_{t}$. Thus, the summation of ${L}_{t}$ over all the $t$ time intervals is equal to ${L}_{project}$. The objective function $\mathcal{J}$ to be minimized is given by:

1 $$\begin{array}{*{20}c} {J = \mathop \sum \limits_{i \in S} \mathop \sum \limits_{t \in T} \left( {F_{i}^{{\text{s}}} s_{i,t} + V_{i,t}^{s} a_{i,t} } \right) + \left( {\mathop \sum \limits_{k \in K} \mathop \sum \limits_{c \in C} \mathop \sum \limits_{t \in T} \beta_{k,c} z_{k,c,t} + \mathop \sum \limits_{k \in K} \mathop \sum \limits_{c \in C} \mathop \sum \limits_{t \in T} \alpha_{k,c} p_{k,c,t} } \right)} \\ { + \mathop \sum \limits_{j \in R} \mathop \sum \limits_{t \in T} \left( {F_{j}^{{\text{r }}} r_{j,t} + F_{j}^{{w{ }}} w_{j,t} + V_{j,t}^{w} b_{j,t} } \right)} \\ \end{array}$$

$\mathcal{J}$ consists of three components, i.e., costs related to CO_2_ capture (the first term), costs related to CO_2_ pipeline construction and transportation (the second term), and costs related to CO_2_ storage (the third term). Specifically, capture and storage costs cover the fixed costs for opening sources, sinks, and wells and the variable costs for capturing and storing one tonne of CO_2_. The definitions of decision and input parameters are presented in Tables 1 and 2.

**Table 1:** Decision parameters in *SimCCS*^*3.0*^.

${s}_{i,t}$	Source node $i$ is open/closed during time interval $t$	Integer, {0, 1}
${r}_{j,t}$	Reservoir node $j$ is open/closed during time interval $t$	Integer, {0, 1}
${w}_{j,t}$	Number of opened wells at reservoir $j$ during time interval $t$	Integer, {0, …, n}
${y}_{k,\mathrm{c},t}$	Pipeline segment $k$ with trend $c$ is open/closed for CO_2_ transport during time $t$	Integer, {0, 1}
${z}_{k,\mathrm{c},t}$	Pipeline segment $k$ with trend $c$ is built or not during time interval $t$	Integer, {0, 1}
${a}_{i,t}$	Annual CO_2_ capture amount at source node $i$ during time interval $t$	Continuous, ${\mathbb{R}}$
${b}_{j,t}$	Annual CO_2_ storage amount at reservoir node $j$ during time interval $t$	Continuous, ${\mathbb{R}}$
${p}_{k,c,t}$	Annual CO_2_ transport flow rate in pipeline segment $k$ with trend $c$ during time interval $t$	Continuous, ${\mathbb{R}}$
${q}_{{\text{CO}}_{2}}$	Annual target CO_2_ captured for this CCS project	Continuous, ${\mathbb{R}}$

**Table 2:** Basic input parameters for CCS pipeline modeling in *SimCCS*^*3.0*^.

${F}_{i}^{s}$, ${F}_{j}^{\text{r}}$, ${F}_{j}^{w}$	Fixed costs for opening source $i$, reservoir $j$, and each well at reservoir $j$
${V}_{i,t}^{s}$, ${V}_{j,t}^{w}$	Variable costs for capturing CO_2_ at source $i$ and injecting CO_2_ at each well at reservoir $j$ at time interval $t$
$S$, $R$, $K$, $C$, $I$, $T$	Set of sources, reservoirs, pipeline arcs, pipeline capacity trends, network nodes, and time intervals
${Q}_{i}^{s}$	CO_2_ capturing rate at source $i$
${Q}_{j}^{w}$	The injection capacity of each well at reservoir $j$
${Q}_{j}^{r}$	Storage capacity of reservoir $j$
${L}_{t}$	Time interval length of time interval $t$
${L}_{project}$	Total CCS project length
${t}^{\mathrm{^{\prime}}}$	Current time interval
${Q}_{k,c}^{max}$, ${Q}_{k,c}^{min}$	Maximum and minimum CO_2_ transport capacities of pipeline segment $k$ with trend $c$
${\alpha }_{k,c}$	Cost of transporting one tonne of CO_2_ using pipeline segment $k$ with trend $c$
${\beta }_{k,c}$	Cost of constructing the pipeline segment $k$ with trend $c$
${\gamma }_{i,t}$	Status indicator of source $i$ at time interval $t$
${TB}_{t}$	Tax benefit for each sink during time interval $t$
$t$	Index of time interval

The constraints for the MIP problem are provided in Eqs. (2)–(12). Specifically, Eq. (2) enforces the flow rate of CO_2_ in one pipeline segment is between its minimum and maximum transportation capacities, respectively, Eq. (3) ensures that flow conservation at each node. In this equation, the subscripts $src$ and $dst$ indicate the inflow and outflow of a certain node, respectively. If the node is a CO_2_ point source (the top row), then the annual flow rate at this node is the CO_2_ capture amount; on the other hand, if this node is a CO_2_ storage site (the middle row), the annual flow rate at this node is annual injection amount. Otherwise, the annual inflow and outflow flow rates of this node are the same (the bottom row). Equation (4) allows at most one linear piece of pipeline to be used. Equation (5) illustrates the CO_2_ capture requires the open status of the source and the capture amount from a given source is below its maximum capacity. Similarly, Eq. (6) ensures that a well must be drilled before injecting CO_2_ and the injected amount is below its injection capacity. Equation (7) ensures that CO_2_ storing amount at a given time interval is less than the storage capacity of the reservoir. It should be noted that although Eqs. (6) and (7) are quite similar, their functionalities are different because Eq. (6) focuses on each individual well injection cap and Eq. (7) focuses on the entire reservoir storage cap. Since these two equations are applied to each time interval, therefore, no summations are required. Equation (8) enforces that the total capture amount from all the sources meets the prescribed emission target. Equation (9) is related to Eq. (7) but it constrains the cumulative CO_2_ stored in the reservoir below the storage capacity. Equation (10) is applied to ensure that only one segment of the pipeline can be built between two notes at most. Equation (11) ensures that the pipeline segment must be built before the starting of transporting CO_2_. This constraint is important because we must make sure that the pipeline is built first then it can be used for CO_2_ transport. No further construction related cost is applied after this pipeline is built.

2 $$Q_{k,c}^{min} y_{k,c,t} \le p_{k,c,t} \le Q_{k,c}^{max} y_{k,c,t} ,\forall k \in K,c \in C,{ }t \in T$$

3 $$\underbrace {{\mathop \sum \limits_{k \in K} }}_{src\left( k \right) = n}\mathop \sum \limits_{c \in C} p_{k,c,t} - \underbrace {{\mathop \sum \limits_{k \in K} }}_{dst\left( k \right) = n}\mathop \sum \limits_{c \in C} p_{k,c,t} = \left\{ {\begin{array}{*{20}l} {a_{n,t} ,} \hfill & {{\text{if }}n \in S} \hfill \\ { - b_{n,t} ,} \hfill & {{\text{if }}n \in R} \hfill \\ {0,} \hfill & {\text{ otherwise }} \hfill \\ \end{array} { }\forall n \in I,\forall t \in T} \right.$$

4 $$\mathop \sum \limits_{{c \in C{ }}} y_{k,c,t} \le 1,\forall k \in K,t \in T$$

5 $$a_{i,t} \le Q_{i}^{s} s_{i,t} { }\forall i \in S,\forall t \in T$$

6 $$b_{j,t} \le Q_{j}^{w} w_{i,t} { }\forall j \in {\text{R}},\forall t \in T$$

7 $$b_{j,t} \le Q_{j}^{r} r_{i,t} { }\forall j \in {\text{R}},\forall t \in T$$

8 $$\mathop \sum \limits_{{i \in S{ }}} a_{i,t} \ge q_{{{\text{CO}}_{{2}} }} ,\forall t \in T$$

9 $$\mathop \sum \limits_{{t \in T{ }}} b_{j,t} L_{t} \le Q_{j}^{r} ,\forall j \in {\text{R}}$$

10 $$\mathop \sum \limits_{c \in C} \mathop \sum \limits_{t \in T} z_{k,c,t} \le 1,\forall k \in K$$

11 $$\mathop \sum \limits_{t = 0}^{{t = t^{\prime}}} y_{k,c,t} \ge \mathop \sum \limits_{t = 0}^{{t = t^{\prime}}} z_{k,c,t} \ge y_{k,t,c}$$

### Impact of the enhanced 45Q tax credits

To consider the enhanced 45Q tax credits in the CCS design optimization problem, we define the tax credit is $${TB}_{t}$$ for time interval $$t$$ with the unit of $/tCO_2_. At a given time interval, this tax credit will be subtracted from the storing cost for each tonne of CO_2_. Therefore, the updated objective function $${\mathcal{J}}^{^{\prime}}$$ can be defined as:

12 $$\begin{array}{*{20}c} {{\mathcal{J}}^{^{\prime}} = \mathop \sum \limits_{i \in S} \mathop \sum \limits_{t \in T} \left( {F_{i}^{{\text{s}}} s_{i,t} + V_{i,t}^{s} a_{i,t} } \right) + \left( {\mathop \sum \limits_{k \in K} \mathop \sum \limits_{c \in C} \mathop \sum \limits_{t \in T} \beta_{k,c} z_{k,c,t} + \mathop \sum \limits_{k \in K} \mathop \sum \limits_{c \in C} \mathop \sum \limits_{t \in T} \alpha_{k,c} p_{k,c,t} } \right)} \\ { + \mathop \sum \limits_{j \in R} \mathop \sum \limits_{t \in T} \left( {F_{j}^{{\text{r }}} r_{j,t} + F_{j}^{{w{ }}} w_{j,t} + \left( {V_{j,t}^{w} - TB_{t} } \right)b_{j,t} } \right)} \\ \end{array}$$

By using this formulation, users can incorporate different scenarios of tax credits into their analysis and evaluations. For the optimization problem presented in this paper, Eq. (12) serves as the objective function. It is worth noting that in our current formulation, the tax credit is fixed for all sinks at a given time. Therefore, the number of tax credit values is the same as the total number of intervals. It is worth exploring the incorporation of spatial and temporal variability of tax credits to model more complex scenarios of the enhanced 45 tax credits in *SimCCS*^*3.0*^, which is currently under development.

### Dynamic evolution of CO_2_ point source status

To model dynamic evolution of CO_2_ point source open/closed status into the optimization problem, it is required to define the CO_2_ point source status indicator ${\gamma }_{i,t}$ for source $i$ at time interval $t$. The value of ${\gamma }_{i,t}$ can only be chosen from 1, 0, and − 1. A value of 1 in ${\gamma }_{i,t}$ indicates that source $i$ is open for CO_2_ capturing, and a value of 0 indicates that the source is closed (no CO_2_ capture). To increase model flexibility and robustness, we also can assign − 1 to ${\gamma }_{i,t}$, which indicates that the status is unknown. Therefore, the optimization algorithm can determine the final open/closed status of source $i$ at interval $t$. To incorporate this parameter into this MIP problem, an additional constraint defined in Eq. (13) is added to the constraint list.

13 $$s_{i,t} = \left\{ {\begin{array}{*{20}l} {1, \,{\text{if }}\gamma_{i,t} = 1,} \hfill \\ {0, \,{\text{if }}\gamma_{i,t} = 0,} \hfill \\ {0\, or\, 1, i{\text{f }}\gamma_{i,t} = 0, } \hfill \\ \end{array} } \right.\forall t \in T$$

**Figure 2:**
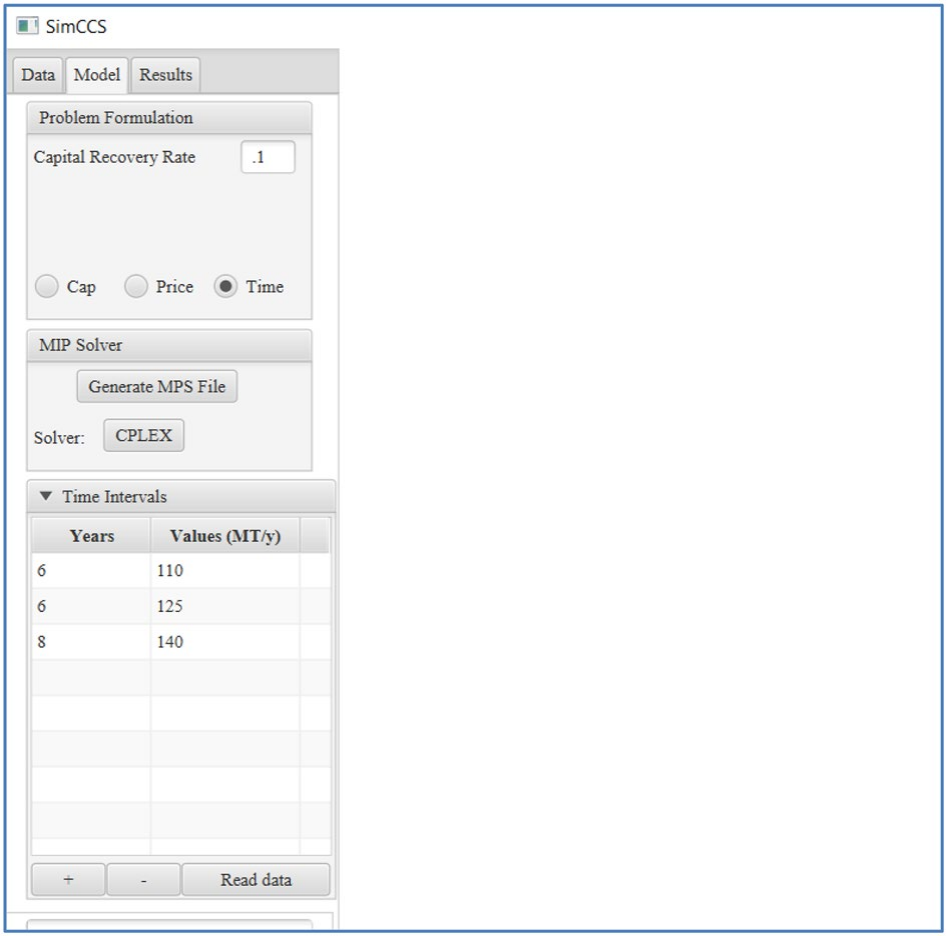
Time mode in the Model panel for *SimCCS*^*3.0*^.

As shown in Eq. (13) and Table 1, ${s}_{i,t}$ is a decision variable and it represents the status of the source node $i$ during time interval $t$, which needs to be determined by an optimizer. On the other hand, ${\gamma }_{i,t}$ is prior information of the status of the source node $i$ during time interval $t$. Users need to provide the values of ${\gamma }_{i,t}$ as input parameters. Otherwise, *SimCCS*^*3.0*^ will assign the default value of − 1 to ${\gamma }_{i,t}$, which results in an automatic determination of source status during the optimization process. For this scenario, ${s}_{i,t}$ can be either 0 or 1 depending on the final optimization solution. The functionality of this constraint is to enforce the open/closed status of source $i$ during time interval $t$ is the same as the predefined status in the input data. By using this constraint, the overall optimization process is simplified because the decision variables are known except the scenario when ${\gamma }_{i,t}$= − 1 $.$ Note that a CO_2_ point source may have different values for $\gamma$ during the entire project life to mimic the dynamic feature CCS technology deployment. For example, ${\gamma }_{i,t}$ can be set as 1, 1, 0, − 1, and 1 for a CO_2_ point source during the five intervals. By using Eqs. (2)–(13), a CCS project design problem can be defined. The next step is to generate an input file for the optimization run. Here we use the Mathematical Programming System (MPS) format to process and store the objective function, decision parameters, constraints, and upper/lower bounds for decision variables. Once the MPS file is ready, it is subjected to an optimizer for searching for optimum solutions. In this work, we perform the optimization run using IBM ILOG CPLEX Optimizer software^29^, which can be run parallelly using multiple CPU cores and threads. The elapsed CPU time for a given optimization run may depend on the nature of a problem, the number of decision variables and constraints, the number of CPU cores used, etc. Some simple problems may only require several seconds to get the final solution. However, it may take up to several days to get solutions for larger or challenging problems. Figure 2 displays the new panel for *Time* mode in *SimCCS*^*3.0*^ to model phase-based CCS infrastructure design.

Once the optimum solution is obtained by running the CPLEX, the result visualization module of *SimCCS*^*3.0*^ can be used to examine the final solution in terms of costs and pipeline routes, diameters, and total lengths. Consider that the paper focuses on the dynamic modeling of CCS infrastructure, therefore, the evolution of costs and pipeline routes and lengths can be provided. The final costs include CO_2_ capturing and storing costs, tax benefits, pipeline construction costs, and CO_2_ transport costs. *SimCCS*^*3.0*^ is a multi-platform tool as it can be run on Windows, Linux, and macOS.

It should be noted that the number of time intervals and the length of each time interval are the important input parameters for the developed phase-based CCS modeling capability. The current modeling capability has great flexibility in choosing the number of intervals and the length for each interval. Users can select the proper number of intervals based on their project requirements.

## Case studies

In this section, we present two case studies to demonstrate the newly developed phase-based modeling feature in *SimCCS*^*3.0*^ including a relatively small case focused on Wyoming and a regional case study focused on the I-WEST region. Note that the information presented in two case studies may not necessarily reflect true data because these case studies are hypothetical and are designed to demonstrate the modeling capabilities of *SimCCS*^*3.0*^. We used an HP laptop with an Intel® Core® i7-1185G7 CPU @ 3.00 GHz and 32 GB RAM to perform all the optimization runs.

### Wyoming case

**Figure 3:**
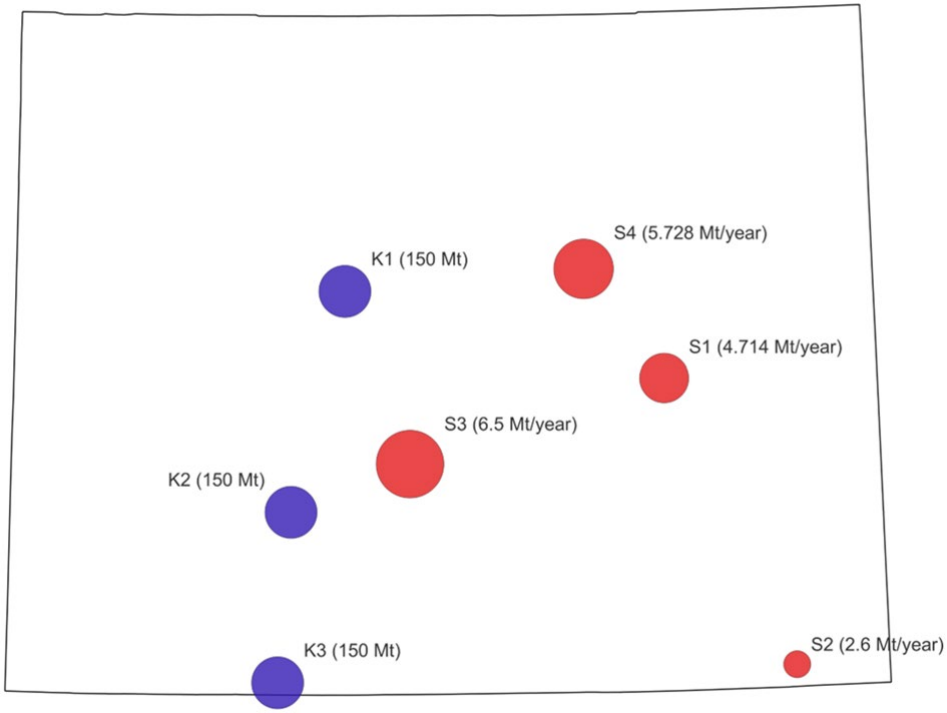
A map of CO_2_ sources and sinks for the Wyoming case study. The blue circle indicates the location of each sink while the size indicates the storage capacity in Mt CO_2_; the red circle denotes the location of each CO_2_ point source while the size indicates the capture amount in Mt/year.

The case focuses on a small-scale CCS deployment project in Wyoming state, and it involves four CO_2_ point sources and three CO_2_ geologic storage sites. The locations of sources and sinks are presented in Fig. 3. The CO_2_ capture or emission amount for the four sources varies from the minimum value of 2.6 Mt/year in S2 (lower right corner) to the maximum value of 6.5 Mt/year in S3. The CO_2_ storage capacity for each sink is assumed to be 150 Mt.

We fix the CO_2_ capture cost as $56/tCO_2_ for all four sources and set the storage cost as $5/tCO_2_. A total of six case runs are performed to account for various scenarios. The detailed input data are presented in Table 3. The overall project is for 20 years for all the six cases, among which, the project life of Cases 1 to 3 is divided into two time intervals to investigate the impacts of the enhanced 45Q tax credits, Cases 4 to 6 into four time periods to demonstrate the dynamic evolution of CO_2_ point sources status. The annual CO_2_ capturing target for Cases 1 to 3 increased from 15 to 19 Mt/year for the two intervals. For Cases 4 to 6, we use different annual CO_2_ capturing targets to test the different scenarios of dynamic evolution of source status.

**Table 3:** Input data for different scenarios for the case study.

	Storage cost ($/tCO_2_)	45Q tax credits ($/tCO_2_)	Capture targets (Mt/year)	Number of time intervals	Time intervals (years)
Case 1	5	[50, 0]	[15, 19]	2	[8,12]
Case 2	5	[50, 50]	[15, 19]	2	[8,12]
Case 3	5	[100, 0]	[15, 19]	2	[8,12]
Case 4	5	[50, 50, 0, 0]	[19.5, 13.5, 16.5, 5.8]	4	[6, 6, 4, 4]
Case 5	5	[50, 50, 0, 0]	[14.5, 2.5, 10, 4.5]	4	[6, 6, 4, 4]
Case 6	5	[50, 50, 0, 0]	[13, 6.6, 12, 6.8]	4	[6, 6, 4, 4]

#### Enhancement in 45Q tax credits

**Figure 4:**
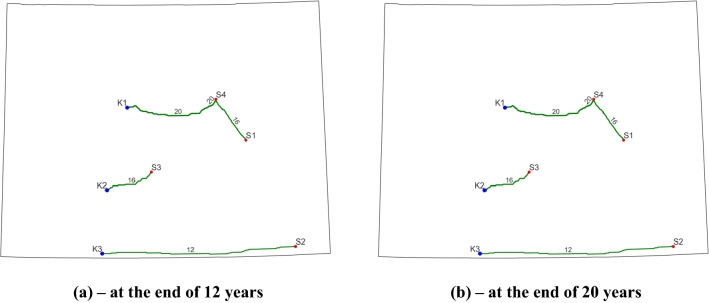
Final optimized CO_2_ pipeline network for Cases 1–3. Blue dots represent sinks, red dots represent CO_2_ point sources, and green curves indicate the optimized pipeline routes (the number and thickness denote the pipeline diameter).

As shown in Table 3, we specify the tax credit for the first interval as 50$/tCO_2_^3^ and 0$/tCO_2_ for the second interval for Case 1. This model setup is corresponding to the 12-year of 45Q tax credits for CO_2_ storage^4^. Again, it is worth noting that this study was conducted before the new 45Q tax credit information under Inflation Reduction ACT of 2022. Figure 4 presents the final optimized pipeline routes at the end of two stages for Cases 1–3. Note that the final optimized CO_2_ transport pipeline networks for Cases 1–3 are the same in this study. Clearly, all the four CO_2_ point sources and three sinks are used in this case. During the first interval of 12 years, the CO_2_ capture amounts for the four sources are 0.172, 2.6, 6.5, and 5.728 Mt/year from S1, S2, S3, and S4. When compared with CO_2_ capture capacities in Fig. 3, the full capacities of S2, S3, and S4 have to be used to meet the predefined capture goal of 15 Mt/year. Only a small portion of CO_2_ capture capacity is used in S1 (0.172 versus 4.714 Mt/year). During the second time interval, because the defined capture target reaches 19 Mt/year, all the CO_2_ sources with 100% capture capacity are required. Regarding the sinks, K1 stores 70.8 and 79.2 Mt/CO_2_ during the two time intervals, which corresponds to its maximum storage capacity. All the CO_2_ captured at S1 and S4 is stored at this site. On the other hand, K2 and K3 only store 130 and 52 Mt CO_2_, respectively. This observation illustrates that it is more cost-effective to use K1 as a storage site for this project.

There are 8 pipeline segments with three different pipeline sizes including 12-, 16-, and 20-inch are built to transport the captured CO_2_ to the sinks. Due to the small amount of CO_2_ captured and transported within the pipelines for this case study, larger pipeline sizes, e.g., 42-inch, are not required. The pipeline routes stay the same for two stages. A careful examination of Fig. 4 reveals that the pipeline sizes increase from 16-inch to 20-inch from S1 to K1. This can be attributed to the increased CO_2_ amounts from S1 to S4 and S4 to K1. A larger pipeline size is required to transport captured CO_2_ from both S1 and S4. Table 4 presents a summary of this case study. A total of 722.39 km of pipelines is needed for this project. In terms of the costs, the total cost for pipeline construction is about $631.24 million and this capital cost is only needed during the first-time interval because no new pipeline construction occurred during the second phase.

**Table 4:** Summary of the final optimization solutions for Cases 1–3.

Cases	Intervals	Total pipeline construction cost (million $)	Total pipeline length (km)	Annual capture cost ($/tCO_2_)	Annual storage cost ($/tCO_2_)	Annual tax credit ($/tCO_2_)
Case 1	T1	631.24	722.39	56	5	50
	T2	0	0	56	5	0
Case 2	T1	631.24	722.39	56	5	50
	T2	0	0	56	5	50
Case 3	T1	631.24	722.39	56	5	100
	T2	0	0	56	5	0

The tax credit result for this case is also presented in Table 4. During the first interval, the annual tax credit received is $750 million. This tax benefit would encourage operators to store more CO_2_. On the contrary, the operators will not receive any tax benefit during the second stage, which results in about $95 million for net CO_2_ storage cost (the difference between storage cost and 45Q tax credits) per year for the following 8 years.

Cases 2 and 3 are two variants of Case 1 to test different scenarios of 45Q tax credits. Case 2 differs from Case 1 only in terms of the 45Q tax credit for the second phase. Specifically, we also provide a $50 tax credit for the remaining 8 years of the CCS project. Case 3 differs from Case 1 by increasing the 45Q tax credit from $50 to $100 per tonne of CO_2_ for the first stage. Because all other input parameters are the same as in Case 1, it is anticipated that the final optimization solutions from Cases 2–3 are the same as Case 1, which is demonstrated by the final pipeline routes in two stages presented in Fig. 4. This observation illustrates that the final pipeline routes depend on not only factors related to CO_2_ storage, but also other factors related to CO_2_ capture and transport.

The results presented in Table 4 and Fig. 4 provide a comprehensive economic analysis of CCS projects. For example, using 50 $/tCO_2_ of tax credit for the second phase in Case 2, suggest that the operators can obtain about $750 million tax credit for 12 years during the first interval and about $950 million tax credit per year for the remaining 8 years. In terms of Case 3, the operators can receive double tax benefits as that in Case 1 during the first stage project. Specifically, the annual tax benefit is $1.5 billion for 12 years, which corresponds to $18 billion for the entire project. This is a large amount of tax credit though there is no 45Q tax credit during the second phase of this project.

As shown in the Eqs. (1) and (12), the objective function consists of three cost components, including CO_2_ capture cost, transport cost, and storage cost. For this case study, the CO_2_ capture and storage cost are assumed to be 56$/tCO2 and 5$/tCO_2_, respectively. As expected, these values are the same as the model inputs presented in Table 3. The CO_2_ transport cost is about 1.15 and 0.7 $/tCO_2_ during this first and second phase, respectively. It is apparent that the cost for CO_2_ capture is the major cost for a CCS project followed by the costs for CO_2_ storage and transport based on this case study.

#### Dynamic evolution of point source open/closed status

Here, we use Cases 4–6 to test the consideration of dynamic evolution of CO_2_ point source status. The total annual CO_2_ capture target is also presented in Table 3. For example, for Case 5, the annual CO_2_ capture target is 19.5, 13.5, 16.5, and 5.8 Mt/year for each four intervals, which are different than that in Cases 1–3. Table 5 presents the open/closed status indicator for each source at each time interval. Because these values are input parameters for the dynamic model in *SimCCS*^*3.0*^, therefore we have to assume that they are known as prior information. Those values represent in Table 5 provide some important insights into practical deployments of CCS projects.

**Table 5:** Input data for different scenarios of evolution of CO_2_ point sources (1 indicates open, 0 indicates closed, and − 1 indicates unknown status).

Case	Source	T1 (6)	T2 (6)	T3 (4)	T4 (4)
Case 4	S1	1	1	1	1
	S2	1	1	0	0
	S3	1	1	1	− 1
	S4	1	0	1	0
Case 5	S1	0	0	1	1
	S2	1	1	0	0
	S3	1	0	0	0
	S4	1	0	1	0
Case 6	S1	0	0	1	1
	S2	1	1	1	0
	S3	1	0	0	0
	S4	− 1	− 1	− 1	− 1

**Figure 5:**
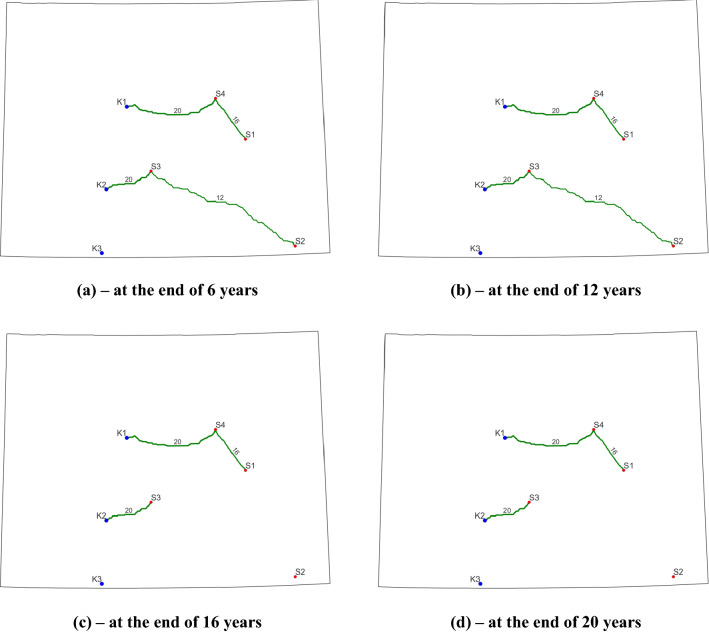
Final optimized CO_2_ pipeline network for Case 4. Blue dots represent sinks, red dots represent CO_2_ point sources, and green curves indicate the optimized pipeline routes (the number and thickness denote the pipeline diameter).

For all three cases, the final solutions provide an annual capture cost and annual storage cost of 56 and 5 $/tCO_2_ for the four phases. In terms of 45Q tax credits, the operators will receive 50 and 0 $/tCO_2_ for the first 12 years and the remaining 8 years, respectively. Figure 5 displays the evolution of pipeline routes and CO_2_ capture and storage data during four stages for Case 4. Similar to Cases 1 to 3, three types of pipeline diameters are used in this case. One interesting observation from Fig. 5 is that all the pipelines are built and used during the first interval and no new construction occurred in the following years. The reason is that during the first 6-year period (T1), the capture target is 19.5 Mt/year, which is the largest capture amount among all the four phases. Therefore, we must build enough pipelines to transport 19.5 Mt CO_2_ annually. The built pipeline network during T1 is sufficient to transport CO_2_ for the following phases (T2-T4). Clearly, we can observe some built pipelines are not used during the ensuing phases. For example, during the third and last interval, the 12-inch pipeline that connects S2 and S3 is not utilized because there is no CO_2_ captured from S2.

Finally, this project requires a total of 691.88 km of pipelines, which is about 95.6% of those in previous cases, and the corresponding construction cost is about $641.83 million (see Table 6). The reason for the reduced pipeline length is that the 12-inch pipeline connecting S2 and K3 used previously is not constructed here. However, we can observe a slight increase in construction costs from $631.24 million to $641.83 million when compared to Cases 1–3. That is because a 20-inch pipeline (more expensive) to connect S3 and K2 is built as in Fig. 5. Remember that in previous cases, a 16-inch pipeline is sufficient to meet the transport target between these two locations (Fig. 4). In terms of storage sites, K3 is not utilized here. One possible reason for this observation is that the annual CO_2_ capture target is much smaller in this case, which results in less amount of CO_2_ required for storage than that in the previous cases (287.2 Mt versus 332 Mt). Thus, to save overall project cost K3 is not operated for CO_2_ storage. By the end of project life, the capacity of K2 (150 Mt) is fully utilized while 137.2 Mt of CO_2_ is stored in K1, which takes about 91.5% of its capacity.

**Table 6:** Summary of the final optimization solutions for Cases 4–6.

Cases	Intervals	Total pipeline construction cost (million $)	Total pipeline length (km)
Case 4	T1	641.83	691.88
	T2	0	0
	T3	0	0
	T4	0	0
Cases 5–6	T1	557.15	600.83
	T2	0	0
	T3	84.68	91.05
	T4	0	0

Figure 5 also reveals important information regarding the open/closed status of each source of the final solution. During T1, all the sources are capturing CO_2_ to meet the capture target of 19.5 Mt/year. This result confirms the prescribed source status defined in Table 5 for T1. We can see that all four sources are in open statuses (“1” in Table 5). During T2 and T3, we open all the sources except S4 and S2, respectively. As expected, the optimum solution only uses S1-S3 for T2 and S1, S3, and S4 for T3 for CO_2_ capture, respectively. The last interval is slightly different as we are not sure whether to open or close S3 (“− 1” in the table). The only information we know about the project is that S1 must be open while S2 and S4 must be closed. To meet the capture target of 5.8 Mt/year, both S1 and S3 are required to open for capture according to the optimized solution. That is because S1 is only capable of capturing a maximum of 4.714 Mt/year (Fig. 3), which is less than the target. Therefore, the optimizer will automatically open S3 to capture a certain amount of CO_2_ to meet this prescribed capturing target. The results of Case 4 demonstrate the feasibility and effectiveness of the developed dynamic modeling for CO_2_ point source status evolution.

To include more practical scenarios, another two case studies, i.e., Case 5 and Case 6 are designed and tested. In Case 5, for example, S1 is not capturing CO_2_ until T3. This is a realistic scenario for the new construction of CO_2_ capture facilities. Though the CCS project is deployed much earlier, some capturing facilities are only available after a certain time. S2 starts capturing CO_2_ from year 1 to year 12. Similarly, S3 only operates during T1 for a total number of 6 years. These settings indicate that S2 and S3 may be coal power plants as there is a trend that coal power plants are phased out gradually. For Case 6, S1 may represent a new facility starting operation during T3. S2 and S3 may be coal power plants and will be shut down after 16 and 6 years, respectively. Once again, S4 works as a backup source to meet defined capture targets for four intervals.

**Figure 6:**
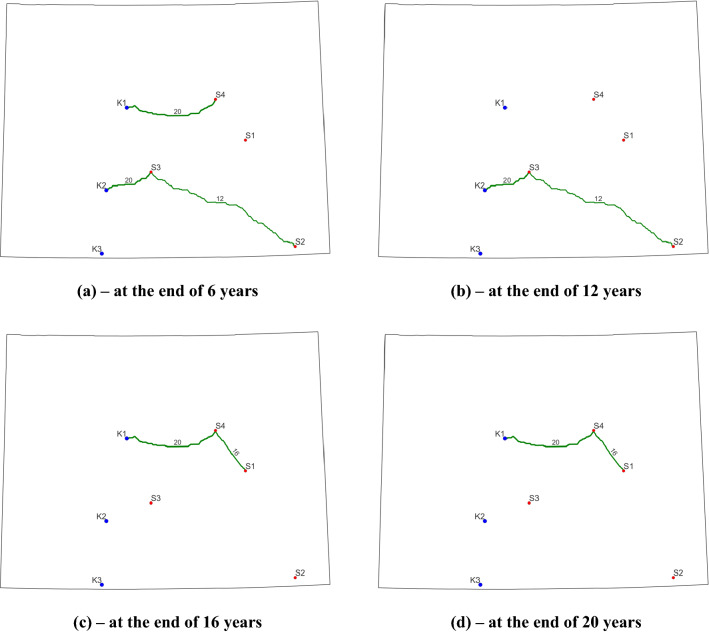
Final optimized CO_2_ pipeline network for Case 5. Blue dots represent sinks, red dots represent CO_2_ point sources, and green curves indicate the optimized pipeline routes (the number and thickness denote the pipeline diameter).

**Figure 7:**
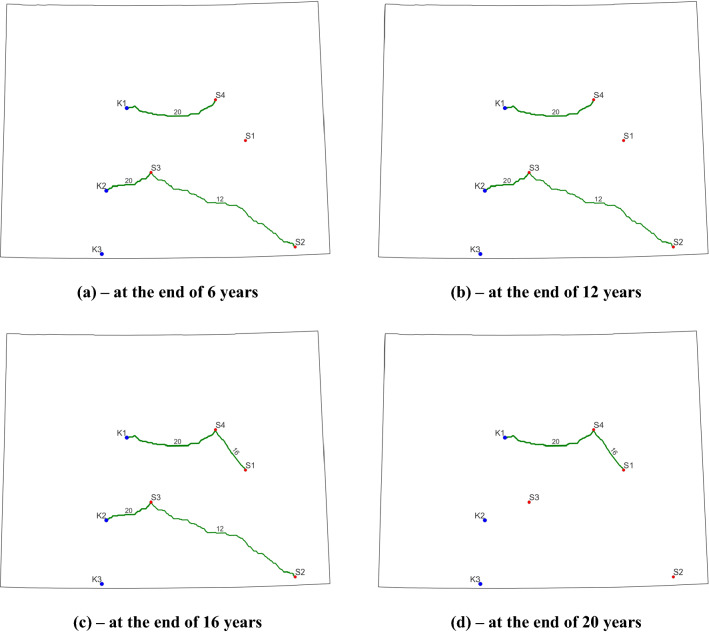
Final optimized CO_2_ pipeline network for Case 6. Blue dots represent sinks, red dots represent CO_2_ point sources, and green curves indicate the optimized pipeline routes (the number and thickness denote the pipeline diameter).

The evolutions of pipeline routes for Case 5 and Case 6 are displayed in Fig. 6 and Fig. 7, respectively. Although there are differences in the pipeline routes between the two cases from interval to interval, a clear examination of those two plots suggests that the final contracted pipelines are identical in terms of locations, diameters, and lengths. This is also confirmed by the pipeline construction information presented in Table 6. The differences in pipeline layout during a certain interval are due to the disparity in annual CO_2_ capture targets and open/closed statuses of sources. During T2, for example, only S2 is operating and capturing 2.5 Mt CO_2_ per year in Case 5. However, during the same period, S2 and S4 are open to capture CO_2_ and the total annual amount is 6.6 Mt in Case 6. Therefore, to transport the captured CO_2_ at S4, we must operate the 16-inch pipeline that connects S4 and K1. For both cases, the operators are required to build 600.83 km pipelines with diameters of 12- and 20-inch during the first 6 years. This new construction requires about $557.15 million. The next new construction occurs during T3 for a 16-inch pipeline connecting S1 and S4. The length and construction cost for this pipeline is 91.05 km and $84.68 million, respectively.

In both cases, CO_2_ is injected into two storage sites including K1 and K2. The cumulative CO_2_ stored at K1 and K2 is 92.37 Mt and 67.63 Mt for Case 5 and 137.7 Mt and 55.1 Mt for Case 6. Apparently, neither of the two storage sites is fullly used here. Regarding the dynamic evolution of source open/closed statuses, the optimized solutions for both cases honor all the prescribed status values in Table 5, which demonstrates the robustness of this model capability. For example, in Case 6, we know that S1 and S2 are open, S3 is closed, and S4 is under an unknown status during T3 as in Table 5. The optimum solution shows that S1, S2, and S4 are capturing about 4.714, 1.558, and 5.728 Mt/ year. It is evident that S3 is not used here and S1 and S2 are opened. To meet the capture target of 12 Mt/year, S4 is also utilized here because the total capacity of S1 and S2 is below the target (10.442 Mt/year versus 12 Mt/year).

### I-WEST case

**Figure 8:**
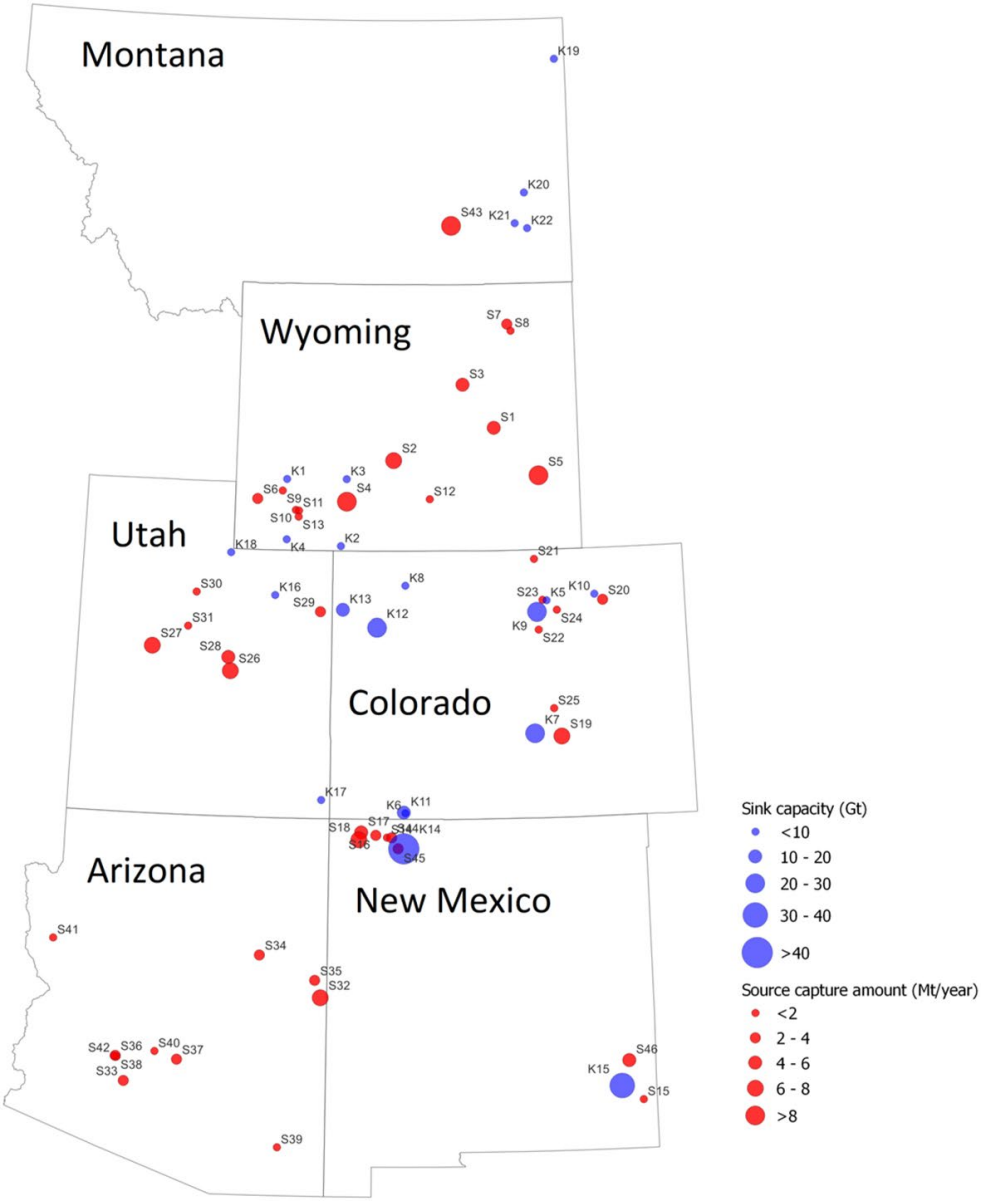
A map of CO_2_ sources and sinks for the I-West case study. The blue circles indicate the locations and storage capacities of CO_2_ storage sinks in Gt CO_2_ while the red circles denote the locations and CO_2_ capture amounts in Mt/year for CO_2_ point sources.

**Figure 9:**
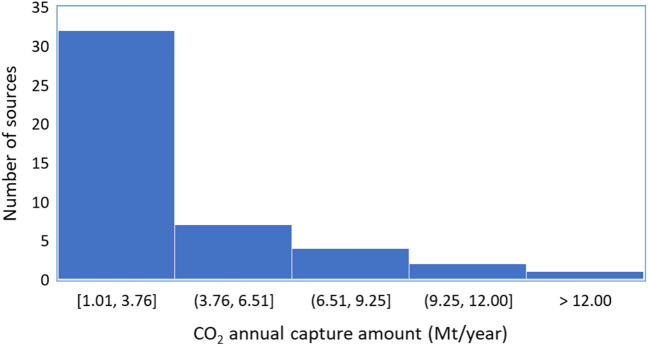
Distribution of annual CO_2_ capture amounts for 44 sources for the I-West case study.

Here we design the CCS infrastructures in the I-WEST region, which contains six states, i.e., Arizona, Colorado, New Mexico, Montana, Utah, and Wyoming. In this case study, the number of storage sinks is 22 and the number of CO_2_ sources is 46^30^. The locations of those sources and sinks are presented in Fig. 8. Though there is at least one CO_2_ source in each state of the I-WEST region, Wyoming has the maximum number of sources, which is followed by Arizona. Only one CO_2_ source, i.e., S43, with a CO_2_ capturing capacity of over 12.751 Mt/year is located in Montana, which is the highest CO_2_ capture amount among all the 46 sources. The source with the lowest CO_2_ capture amount is S14 in Texas. The average capturing capacity for 46 sources is 3.5 Mt/year. Figure 9 shows the distribution of annual CO_2_ capture amounts for this case study. It is easy to notice that 32 of the 46 sources capture below 3.76 Mt/year CO_2_. The number of sources declines as the capture amount increases, which is probably due to the actual difficulties in building large CO_2_ capture facilities. Unlike in the first case study where the capture cost for each source is fixed, we use different values including 14, 53, 56, and 75 $/tCO_2_. Specifically, the costs of three sources are set as 14 $/tCO_2_, two sources as 53 $/tCO_2_, 26 sources as 56 $/tCO_2_, and the remaining 15 sources as 75 $/tCO_2_. This setup would mimic the actual varying CO_2_ cost from different types of facilities.

In terms of sinks, the locations of 22 sinks are also not uniformly distributed across the I-WEST region. It is easy to notice that no CO_2_ storage site is constructed in Arizona despite a large number of CO_2_ sources. Therefore, it is anticipated that CO_2_ captured in Arizona will be transported to other states, e.g., New Mexico, or Utah. The storage capacities are also presented in Fig. 8. The maximum, minimum, and average storage capacities are 50.194, 0.11, and 9.14 GT, respectively. In this case, the storage cost for all the 22 sites is fixed as 5 $/tCO_2_.

Again, this CCS project in the I-WEST region case study will last 20 years. To demonstrate the newly developed dynamic features in the *SimCC*^*3.0*^ tool, we divided the 20-year projects into three-time intervals, including T1, T2, and T3 for 6, 6, and 8 years, respectively. To be consistent with Section 45Q of the U.S.^3^, the tax credits for storing CO_2_ are set as 50, 50, and 0 $/tCO_2_ for three intervals. The evolution of open/closed status input data of all sources is provided in Table 7. In this case, we assume the majority of sources are open, for example, S1 to S8 and S40 to S46. Only several sources including S9, S14, S17, S19, S20, S28, S33, and S39 have more than one status, such as closed, unknown, or both. In this case, the annual capture targets are 110, 125, and 140 Mt/year for three intervals. The increased CO_2_ capture amount is to mimic the trends of the decarbonization process.

**Table 7:** Input data for different scenarios of evolution of CO_2_ point sources for the I-WEST case study (1 indicates open, 0 indicates closed, and − 1 indicates unknown status).

Source	Dynamic evolution of CO_2_ point source statue			Source	Dynamic evolution of CO_2_ point source status		
	T1 (6)	T2 (6)	T3 (8)		T1 (6)	T2 (6)	T3 (8)
S1	1	1	1	S24	1	1	1
S2	1	1	1	S25	1	1	1
S3	1	1	1	S26	1	1	1
S4	1	1	1	S27	1	1	1
S5	1	1	1	S28	− 1	− 1	0
S6	1	1	1	S29	1	1	1
S7	1	1	1	S30	1	1	1
S8	1	1	1	S31	1	1	1
S9	0	1	1	S32	1	1	1
S10	1	1	1	S33	1	− 1	1
S11	1	1	1	S34	1	1	1
S12	1	1	1	S35	1	1	1
S13	1	1	1	S36	1	1	1
S14	0	0	1	S37	1	1	1
S15	1	1	1	S38	1	1	1
S16	1	1	1	S39	− 1	1	1
S17	1	0	1	S40	1	1	1
S18	1	1	0	S41	1	1	1
S19	1	0	0	S42	1	1	1
S20	1	0	1	S43	1	1	1
S21	1	1	1	S44	1	1	1
S22	1	1	1	S45	1	1	1
S23	1	1	1	S46	1	1	1

**Figure 10:**
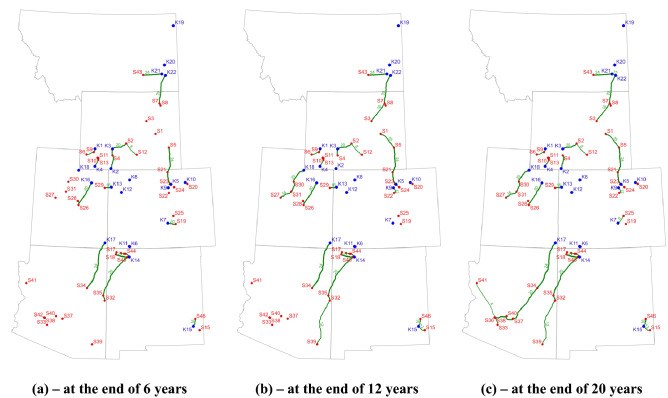
Evolution of pipeline construction for the I-West case: (**a**) at the end of year 6; (**b**) at the end of year 12 and (**c**) at the end of year 20. The green curve denotes the optimized pipeline route, and the thickness of the curve and the number on the curves represent the pipeline sizes (diameter); the red dots represent source location, while the blue dot indicates sink location.

The optimized pipeline routes are presented in Fig. 10. Apparently, from the evolution of pipeline networks, we see that as the CCS project continues, more pipelines are constructed to transport CO_2_. During T1, several major pipelines are built, including, e.g., the 20-inch pipeline connecting K12 and S32 and the 24-pipeline connecting K17 and S34. Next, we must build additional pipelines during T2, including, e.g., the 12-inch pipeline connecting S39 and S32. Finally, during the last interval, several pipelines are constructed to connect most of the point sources, e.g., those sources within the southwest part of the I-WEST region. The dynamic behavior of the pipeline infrastructure is also confirmed in Table 8. This table indicates the evolution of pipeline constructions over time. The initial pipeline construction is 2049.80 km during the first 6 years (T1), which is followed by an additional 891.18 km during the second interval. Finally, we have to build about 732.64 km of new pipelines to accommodate CO_2_ transportation during T3. Therefore, the total pipeline length of this project is 3673.65 km with a total cost of $4.03 billion.

**Table 8:** Summary of final optimization solution for the I-WEST case study.

Intervals	Total pipeline construction cost (billion $)	Total pipeline length (km)	Annual capture cost ($/tCO_2_)	Annual storage cost ($/tCO_2_)	Annual tax credit ($/tCO_2_)
T1	2.65	2049.83	54.66	5	50
T2	0.76	891.18	54.88	5	50
T3	0.62	732.64	57.33	5	0

**Figure 11:**
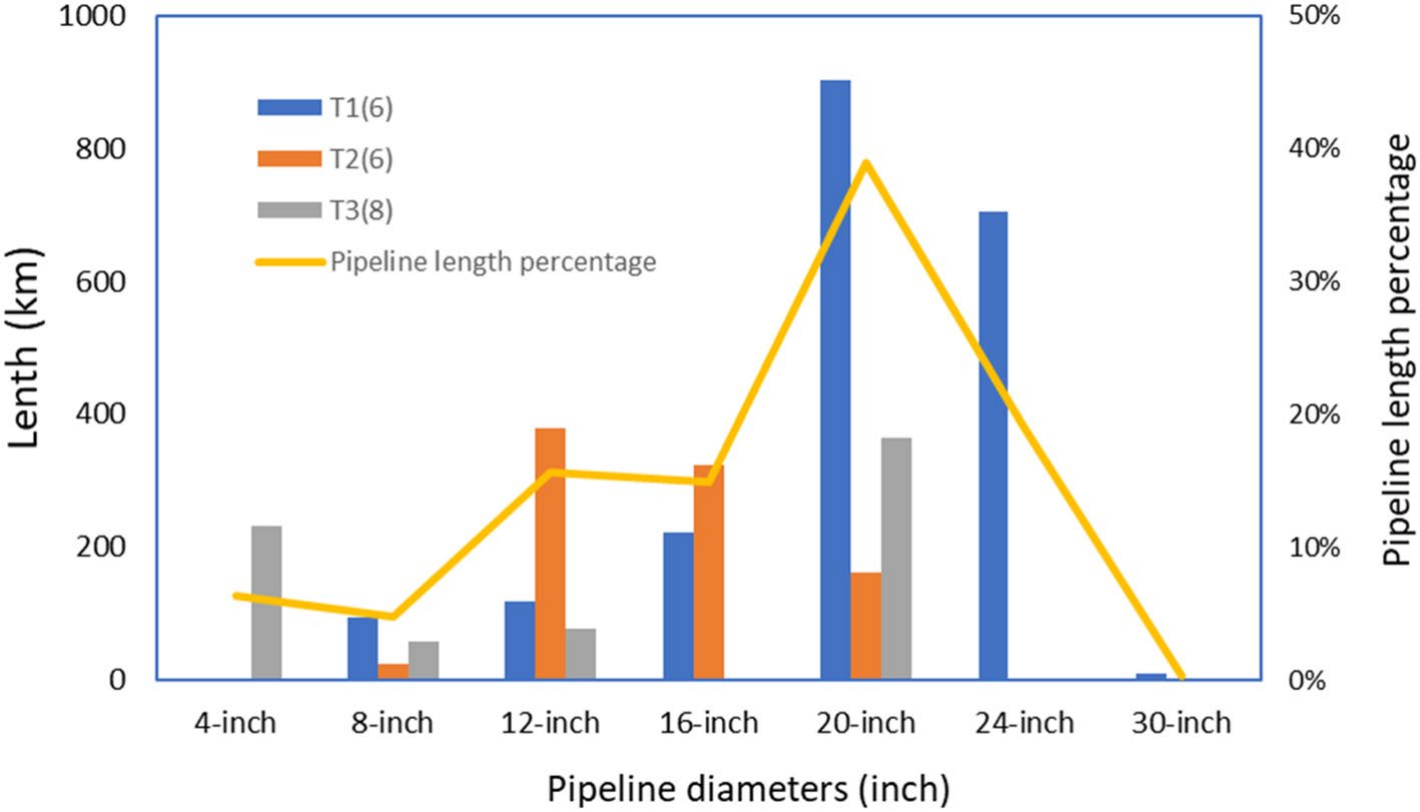
Lengths and diameters of the constructed pipeline for three intervals from the optimized solution for the I-WEST case study.

Figure 11 presents the diameters of newly constructed pipelines together with the corresponding lengths over the project life. Unlike the previous case study, where only three types of pipelines are used, here, pipelines of seven diameters are utilized. For example, a segment of a 10.03 km 30-inch pipeline (0.273% of the total pipeline length) is constructed between S45 and K14 in northern New Mexico during the first interval. Note that the 30-inch pipeline can transport a maximum of 35.13 Mt CO_2_ per year. Figure 11 also reveals that the 20-inch pipeline is mostly used, and it takes about 38.886% of the total pipeline length, which is followed by the 16-inch pipeline for about 547.51 km (about 14.904% of the total pipeline length). Another interesting observation is that we need to build around 232.08 km 4-inch pipeline to connect S41 to the S36 in this case. Although the maximum capacity of a 4-inch pipeline is only about 0.19 Mt/year, it is sufficient to transport the captured 0.175 Mt CO_2_ at S4 per year.

Although 22 sinks are available for CO_2_ storage, only 16 sinks are used in this case study. Among those used sinks, there are large disparities in cumulative CO_2_ stored. Specifically, K14 stores the maximum amount of CO_2_ for 536.21 Mt for 20 years, followed by K5 and K21 for 306.4 and 273.91 Mt CO_2_, respectively. On the other hand, K9 in Colorado stores the smallest amount of CO_2_ for only 12.97 Mt. Table 8 displays the annual storage cost is $5 per tonne of CO_2_ for 20 years. However, the tax credit is $50 per tonne of CO_2_ during the first 12 years and zero for the last interval. The actual tax benefit received during the first and second intervals reaches $70.5 billion.

The optimum solution shows that 25 sources are operating to capture 110 Mt CO_2_ per year during the first interval and the corresponding capturing cost of $54.66 per tonne. Five more sources are used during the second interval to meet the capture target of 125 Mt/year and a comparable capture cost of $54.88 per tonne is suggested. Finally, a total of 43 sources are open to capturing 140 Mt/year with a slightly higher capturing cost of $57.33 per tonne CO_2_. This observation suggests that to meet a higher capture target, more sources are required for capturing CO_2_.

We now examine the open/closed statuses of CO_2_ point sources in Fig. 10. It is evident that S14 is closed until the beginning of T3, which is consistent with the input setting in Table 7. Similar evolution behavior can also be observed for S9, which is closed during T1. These sources can be treated as newly built facilities and often start operating at later stages. A typical coal power plant can be represented as S19, which is operating during T1 and its CO_2_ capturing amount is 7.37 Mt/year. After operating for 6 years, S19 is shut down for the following years of the CCS project. Therefore, there is no CO_2_ is captured from this source, which again agrees with our initial setting in Table 7. It is worth noting that some sources may still not capture CO_2_ despite their “open” statuses. For example, as indicated in Fig. 10, S41 only captures CO_2_ during T3. However, its source status is “1” for all the intervals in Table 7. This behavior can be easily explained though it looks abnormal. Each source is controlled by both the open/closed status (${s}_{i,t}$) and the actual capturing amount (${a}_{i,t}$). Despite it is in open status defined in the input table and as also determined by the optimizer, S41 does not need to capture any amount of CO_2_ because of the small CO_2_ capture targets for the overall project for T1 and T2.

## Conclusion

In this study, we developed and presented a new phase-based modeling capability in the latest *SimCCS*^*3.0*^ tool. Rather than only using a one interval, the entire CCS project life can be divided into multiple periods, which enables users to dynamically model different practical operation scenarios for CCS project deployments. By using the developed dynamic models, we added two novel features in *SimCCS*^*3.0*^ software, including the considerations of the enhancement of 45Q tax credits and dynamic evolutions of source status. These two new capabilities are not only capable of providing the optimized CO_2_ capture, transportation, and storage infrastructure but also giving users freedom to model different practical CCS operational scenarios.

Our results using the Wyoming and the I-WEST case studies indicate that the consideration of 45Q tax credits may not directly impact the final pipeline routes, lengths, and sizes. But it would affect the financial benefits or cost savings for CO_2_ storage. Apparently, a higher 45Q tax credit results in a higher benefit received by the operators. Given the fact that the 45Q tax credits are available for up to 12 years, the operators can benefit significantly from the earlier phases of CCS operations. The dynamic evolution of CO_2_ point source statuses provides users a chance to incorporate their prior information of CO_2_ capturing facilities into the optimization process. The new functionality facilitates the optimizer to exclude those sources from capturing CO_2_ if they are closed during certain intervals and vice versa. Thus, users can model more complex scenarios of dynamic statuses for CO_2_ captures.

Despite these promising results from dynamic models, there is abundant room for further development and applications of the *SimCCS*^*3.*^ tool. First, the consideration of environmental and justice restrictions in CCS infrastructure design should be explored to reduce the environmental burdens on disadvantaged communities. Second, it would be beneficial to utilize some existing CO_2_ pipelines and/or rights-of-way for CO_2_ transportation infrastructure design, which would significantly save the overall cost of the CO_2_ project deployment. Finally, the extension of this design tool for blue hydrogen operations is also worth exploring in the future.

## Data Availability

The datasets used and/or analysed during the current study are available from the corresponding author upon reasonable request.

## References

[1] Chen B (2018). Geologic CO_2_ sequestration monitoring design: A machine learning and uncertainty quantification based approach. Appl. Energy.

[2] Bui M (2018). Carbon capture and storage (CCS): The way forward. Energy Environ. Sci..

[3] Larson, J.B. Committees - H.R.1892 - 115th Congress (2017–2018): Bipartisan Budget Act of 2018. [legislation] 2018 02/09/2018.

[4] Jones, A.C. and M.F. Sherlock, The Tax Credit for Carbon Sequestration (Section 45Q). Congressional Research Service Report, (2020).

[5] Date, R.P., N.O. Asia, and M. East, Global and regional coal phase out requirements of the Paris agreement: Insights from the IPCC special report on 1.5 C. (2019).

[6] Middleton RS (2020). SimCCS: An open-source tool for optimizing CO_2_ capture, transport, and storage infrastructure. Environ. Model. Softw..

[7] Middleton RS (2012). A dynamic model for optimally phasing in CO_2_ capture and storage infrastructure. Environ. Model. Softw..

[8] Middleton RS, Bielicki JM (2009). A scalable infrastructure model for carbon capture and storage: SimCCS. Energy Policy.

[9] Ma, Z., B. Chen, and R. Pawar, An advanced open-source software for the design of CO_2_ capture, transport, and storage infrastructure, in SPE Eastern Regional Meeting. 2022: Wheeling, WV.

[10] Middleton RS, Brandt AR (2013). Using infrastructure optimization to reduce greenhouse gas rmissions from oil sands extraction and processing. Environ. Sci. Technol..

[11] Chen, B. and R.J. Pawar, SimCCS: CCS Infrastructure decision support. (2022).

[12] Keating GN (2011). How storage uncertainty will drive CCS infrastructure. Energy Procedia.

[13] Kim C (2018). Practical deployment of pipelines for the CCS network in critical conditions using MINLP modelling and optimization: A case study of South Korea. Int. J. Greenhouse Gas Control.

[14] Edwards RWJ, Celia MA (2018). Infrastructure to enable deployment of carbon capture, utilization, and storage in the United States. Proc. Natl. Acad. Sci..

[15] Wang Z (2014). Optimal pipeline design for CCS projects with anticipated increasing CO_2_ flow rates. Int. J. Greenhouse Gas Control.

[16] Ma Z, Volkov O, Durlofsky LJ (2022). Multigroup strategy for well control optimization. J. Pet. Sci. Eng..

[17] Awotunde AA (2019). A comprehensive evaluation of dimension-reduction approaches in optimization of well rates. SPE J..

[18] Ma Z, Leung JY (2020). Design of warm solvent injection processes for heterogeneous heavy oil reservoirs: A hybrid workflow of multi-objective optimization and proxy models. J. Petrol. Sci. Eng..

[19] Chen, B. and R. Pawar, Joint optimization of well completions and controls for CO_2_ enhanced oil recovery and storage. *SPE Reserv. Eval. Eng.* 1–12 (2020).

[20] Ma, Z., L. Coimbra, and J. Leung, Design of steam alternating solvent process operational parameters considering shale heterogeneity. SPE Production & Operations, 2022: p. 1–17.

[21] Jin ZL, Durlofsky LJ (2018). Reduced-order modeling of CO_2_ storage operations. Int. J. Greenh. Gas Control.

[22] Johnson N, Ogden J (2011). Detailed spatial modeling of carbon capture and storage (CCS) infrastructure deployment in the southwestern United States. Energy Procedia.

[23] Kuby MJ, Bielicki JM, Middleton RS (2011). Optimal spatial deployment of CO_2_ capture and storage given a price on carbon. Int. Reg. Sci. Rev..

[24] Middleton RS (2020). Great SCO_2_T! Rapid tool for carbon sequestration science, engineering, and economics. Appl. Comput. Geosci..

[25] Meng, M. *et al.* Development and application of advanced sequestration of CO_2_ tool for carbon storage. in *16th Greenhouse Gas Control Technologies Conference 2022 (GHGT-16)*. (Lyon, France, 2022).

[26] Hoover B, Yaw S, Middleton R (2020). CostMAP: An open-source software package for developing cost surfaces using a multi-scale search kernel. Int. J. Geogr. Inf. Sci..

[27] Floudas CA, Lin X (2005). Mixed integer linear programming in process scheduling: Modeling, algorithms, and applications. Ann. Oper. Res..

[28] Ma, Z., Chen, B., Pawar, R. Large-scale CCS infrastructure modeling via SimCCS^3.0^, in *Geological Society of America Abstracts with Programs*. (2022, Denver, CO, USA)

[29] IBM, ILOG CPLEX Optimization Studio. (2021).

[30] Ma, Z., B. Chen, and R. Pawar. Practical consideration of environmental and justice (E&J) in large-scale CCS pipeline infrastructure modeling. in *AGU Fall Meeting Abstracts*. (2022, Chicago, IL, USA)

